# An Efficient Nitroblue Tetrazolium Staining and Bright-Field Microscopy Based Method for Detecting and Quantifying Intracellular Reactive Oxygen Species in Oocytes, Cumulus Cells and Embryos

**DOI:** 10.3389/fcell.2020.00764

**Published:** 2020-08-04

**Authors:** Pradeep K. Javvaji, Arindam Dhali, Joseph R. Francis, Atul P. Kolte, Anjumoni Mech, Sudhir C. Roy, Ashish Mishra, Raghavendra Bhatta

**Affiliations:** ^1^OMICS Laboratory, ICAR-National Institute of Animal Nutrition and Physiology, *Bengaluru*, India; ^2^Center for Post Graduate Studies, Jain University, Bengaluru, India

**Keywords:** intracellular ROS, detection, NBT, oocyte, cumulus cell, embryo

## Abstract

Assessment of intracellular reactive oxygen species (ROS) is important for evaluating the developmental ability of cumulus-oocyte complexes (COC) and embryos. Although, fluorescence-based 2′,7′-dichlorodihydrofluorescein diacetate (DCFH-DA) staining method is used widely for detecting intracellular ROS in COC and embryos, it is associated with several limitations. This study aimed to develop an alternative method for detecting and quantifying intracellular ROS in oocytes, cumulus cells and embryos based on nitroblue tetrazolium (NBT) staining and bright-field microscopy. Nitroblue tetrazolium reacts with ROS and forms formazan precipitate that can be detected as dark purple/blue spots under bright-field microscope. Ovine COC were matured *in vitro* without (control) or with the supplementation of Interleukin-7 (IL-7; for stimulating intracellular ROS), Tempol (superoxide scavenger) or combination of IL-7 and Tempol. The matured COC were stained with NBT and the formation of intracellular formazan precipitates was assessed. Additionally, the matured COC were stained with DCFH-DA to compare the level of intracellular ROS. Further, ovine embryos (8-cell, morula, and degenerating) were generated *in vitro* and stained with NBT for assessing intracellular ROS. The level of intracellular ROS was expressed as the proportion (%) of the NBT stained area of oocytes, compact cumulus cell masses or embryos. The proportions of NBT stained area in the matured oocytes and cumulus cells was found significantly lesser in the control as compared to the IL-7 (1 and 5 ng/ml) treated groups. A similar trend in the intracellular ROS level was also observed in the matured COC, when assessed based on the DCFH-DA staining. Following the treatment with Tempol (100 mM), negligible NBT stained area in oocytes and cumulus cells was observed. The NBT staining patterns of the oocytes and cumulus cells following the combined treatment with IL-7 (5 ng/ml) and Tempol (10 and 25 mM) were comparable with that of the control. The proportion of NBT stained area did not differ significantly between the 8-cell embryos and morula, but was found significantly greater in the degenerating embryos. In conclusion, the developed NBT staining method was found effective for detecting and interpreting the level of intracellular ROS in oocytes, cumulus cells and embryos. This method can be used as an alternative to the DCFH-DA staining method.

## Introduction

Reactive oxygen species (ROS) are the by-products of different metabolic pathways, which are produced by the incomplete reduction of oxygen. They are essential for cellular functioning and their level in cellular system is tightly regulated by the antioxidant defense system. The level of intracellular ROS within the physiological limit is beneficial for cells (Khazaei and Aghaz, 2017). Reactive oxygen species play important roles in ovulation, oocyte maturation, fertilization and embryo development (Siristatidis et al., 2016; Kala et al., 2017). Nevertheless, high level of intracellular ROS induces apoptosis in granulosa cells and oocytes (Khazaei and Aghaz, 2017). Similarly, high intracellular ROS level is associated with poor oocyte maturation, embryonic block and cell death (Bain et al., 2011; Kala et al., 2017). Therefore, most studies focusing the developmental ability and cellular functions of oocytes and embryos include the level of intracellular ROS as an important parameter for better interpretation of results.

The detection of intracellular ROS relies on measuring the end product formed by their reaction with a dye used for the purpose. The end products are detected based on the fluorescence or color they produce (Gosalvez et al., 2017). The most commonly used method for detecting intracellular ROS in oocytes and embryos is the 2, 7′-dichlorodihydrofluorescein diacetate (DCFH-DA) fluorescence assay (Bae, 2015; Mihalas et al., 2017; Song et al., 2017; Yang et al., 2018). Oxidation of DCFH-DA by ROS results in the formation of a fluorescent product DCF that can be detected by fluorescence microscopy (Kalyanaraman et al., 2012). Nevertheless, several limitations are associated with the DCFH-DA assay, such as non-specific oxidation of DCFH-DA (Wardman, 2007; Kalyanaraman et al., 2012), saturation of DCF fluorescence signal (Roesslein et al., 2013) and photobleaching and photooxidation of the DCF-based probes (Choi et al., 2012). Furthermore, the processing and handling of samples for the DCFH-DA staining and documentation of the obtained fluorescence signals are sensitive and cumbersome. Besides, the DCFH-DA assay requires a costly fluorescent microscope. Therefore, the availability of an efficient non-fluorescence technique for assessing intracellular ROS in oocytes, cumulus cells and embryos would be a suitable alternative for the developmental biologists.

Nitroblue tetrazolium (NBT) is a pale yellow color water-soluble nitro-substituted aromatic tetrazolium compound. It is an artificial electron acceptor and NBT assay has been suggested to be a useful tool in the studies of free radicals (Zhang et al., 1993; Esfandiari et al., 2003). The principle of the assay is that NBT enters within the targeted cells following their incubation in NBT solution. Within the cells, the interaction of NBT with superoxide disrupts the tetrazole ring and tetrazoinyl radical is generated, which subsequently dismutates to generate the purple/blue water insoluble and stable formazan crystals (Esfandiari et al., 2003; Volk and Moreland, 2014; Stockert et al., 2018). The reduction product (formazan) can be measured by spectrophotometer or by microscopy (Volk and Moreland, 2014). Previous reports indicate the use of NBT staining for detecting ROS in different biological samples of plant (Dauphinee et al., 2017) and animal (Zhang et al., 1993; Esfandiari et al., 2003; Lagathu et al., 2007; Tunc et al., 2010; Gosalvez et al., 2017) origins. Nevertheless, to the best of our knowledge, currently no report is available on the assessment of intracellular ROS in oocytes, cumulus cells or embryos based on NBT staining.

The current study aimed to develop an efficient alternative method based on NBT staining and bright-field microscopy for detecting and quantifying intracellular ROS in ovine oocytes, cumulus cells, and embryos.

## Materials and Methods

The fetal bovine serum (FBS), gentamicin, M199 Earle’s salt (bicarbonate-buffered M199), M199 Hanks’ balanced salt (HEPES-buffered M199), MEM amino acids solution 50X (EAA) and MEM non-essential amino acids solution 100X (NEAA) were procured from Life Technologies Corporation, NY, United States. The non-fat dry milk powder was procured from Bio-Rad Laboratories Inc., CA, United States and the strepto-penicillin was procured from Cadila Healthcare Ltd., Vadodara, India. The estrous ewe serum was prepared in the laboratory. All other chemicals used in this study were procured from Sigma-Aldrich Co., MO, United States. All experiments including the use of ram for semen collection met the guidelines and regulations of the Institutional Animal Ethics Committee (IAEC) of the ICAR-National Institute of Animal Nutrition and Physiology (ICAR-NIANP), Bengaluru, India.

### Experimental Design

Different experiments were conducted to assess the level of intracellular ROS in ovine oocytes, cumulus cells and embryos based on NBT staining and bright-field microscopy. Cumulus-oocyte complexes (COC) were treated with Interleukin-7 (IL-7), Tempol (4-Hydroxy-2,2,6,6-tetramethylpiperidine 1-oxyl) or combination of IL-7 and Tempol to achieve different level of intracellular ROS in oocytes and cumulus cells. The status of intracellular ROS in COC was also confirmed based on the DCFH-DA staining.

In the first experiment, intracellular ROS in oocytes and cumulus cells was assessed based on the NBT staining. Ovine COC were subjected to *in vitro* maturation (IVM) without (control) or with IL-7 supplementation (1 or 5 ng/ml; 0 to 24 h of IVM). Intracellular ROS was detected and quantified in the matured oocytes and cumulus cells. In the treatment groups, COC were treated with IL-7 to induce greater production of intracellular ROS (Maeurer et al., 2000; Javvaji et al., 2019).

In the second experiment, the effect of Tempol (superoxide scavenger) treatment on intracellular ROS in oocytes and cumulus cells was assessed based on the NBT staining. Ovine COC were matured *in vitro* without (control) or with Tempol supplementation (1, 10, 25, 50, or 100 mM; 19–24 h of IVM). Intracellular ROS was detected and quantified in the matured oocytes and cumulus cells. In the treatment groups, COC were treated with Tempol to neutralize intracellular ROS (Luo et al., 2009).

In the third experiment, the effect of the combined treatment of Tempol and IL-7 on intracellular ROS in oocytes and cumulus cells was assessed based on the NBT staining. Ovine COC were matured *in vitro* without (control) or with the supplementation of IL-7 (5 ng/ml; 0–24 h of IVM) or combination of IL-7 (5 ng/ml; 0–24 h of IVM) and Tempol (10 or 25 mM; 19–24 h of IVM). Intracellular ROS was detected and quantified in the matured oocytes and cumulus cells. Tempol was supplemented in combination with IL-7 to neutralize the IL-7 mediated ROS generation.

In the fourth experiment, intracellular ROS in COC was assessed based on the DCFH-DA staining. Ovine COC were matured *in vitro* without (control) or with IL-7 supplementation (1 or 5 ng/ml; 0 to 24 h of IVM) and intracellular ROS was detected in the matured COC. In the treatment groups, COC were treated with IL-7 to induce greater production of intracellular ROS.

In the fifth experiment, intracellular ROS was assessed in embryos based on the NBT staining. Ovine embryos (8-cell embryos, morula, and degenerating embryos) were generated *in vitro* and subjected to the NBT staining for detecting and quantifying intracellular ROS.

### Collection of COC and IVM

Ovine COC were collected and subjected to IVM as described previously (Javvaji et al., 2020). Briefly, ovine ovaries were collected from a local abattoir and COC were aspirated from the 2–6 mm follicles in aspiration medium (HEPES-buffered M199 supplemented with 50 IU/ml heparin, 50 μg/ml gentamicin and 4 mg/ml fatty acid free BSA fraction V). The COC with more than five layers of compact cumulus cells and homogeneous cytoplasm were collected, washed and matured in B199 medium (bicarbonate-buffered M199 supplemented 0.2 mM sodium pyruvate, 50 μg/ml gentamicin, 0.1 mM cysteamine and 10% FBS) supplemented with 0.01 IU/ml of ovine-FSH, 0.02 IU/ml of human-LH, 1 μg/ml of 17b-estradiol and 10% FBS. The COC were matured *in vitro* for 24 h at 38.5°C in a CO_2_ incubator (5% CO_2_ in a humidified environment). In the treatment groups, the maturation drops were supplemented with IL-7, Tempol or combination of IL-7 and Tempol.

### *In vitro* Embryo Production

Ovine embryos were produced *in vitro* according to the methods described previously (Javvaji et al., 2020). Briefly, COC were matured *in vitro* as described above without any supplementation. On the day of IVF, semen sample was collected from a healthy ram using a handheld electro ejaculator for small ruminants. The neat semen was diluted with warm (37°C) milk-egg yolk extender (1 g of non-fat dry milk powder, 90 mg of glucose, 500 μg of gentamicin, and 1 ml of egg yolk in a total volume of 10 ml) for a final spermatozoa concentration of 50 × 10^6^/ml. The diluted semen sample was stored at 4°C for 4 h and then subjected to percoll gradient method to prepare spermatozoa for IVF.

Following the 24 h of IVM, the COC were washed in SOFH-IVF (107.70 mM NaCl, 20.00 mM HEPES, 7.15 mM KCl, 5.00 mM NaHCO3, 3.32 mM sodium lactate, 1.71 mM CaCl2, 0.33 mM sodium pyruvate, 0.30 mM KH2PO4, 50 μg/ml gentamicin, 5 μg/ml phenol red and 3 mg/ml BSA; pH 7.2 to 7.4) and SOF-IVF (107.70 mM NaCl, 7.15 mM KCl, 25.00 mM NaHCO3, 3.32 mM sodium lactate, 1.71 mM CaCl2, 0.33 mM sodium pyruvate, 0.30 mM KH2PO4, 50 μg/ml gentamicin, and 5 μg/ml phenol red; pH 7.2 to 7.4) media and 10–12 COC in 10 μl of SOF-IVF medium were transferred to 38 μl drops of SOF-IVF medium (supplemented with 5% estrous ewe serum). Subsequently, 2 μl of prepared spermatozoa was added to each drop for a final volume of 50 μl and final spermatozoa concentration of 1.5 × 10^6^/ml. The sperm-oocyte incubation was performed at 38.5°C for 24 h in a CO_2_ incubator (5% CO_2_ in a humidified environment).

At the end of the sperm-oocyte incubation, presumptive zygotes were stripped off cumulus cells, washed and cultured in 20 μl drops of SOF-IVC (107.70 mM NaCl, 25.07 mM NaHCO3, 7.16 mM KCl, 3.30 mM sodium lactate, 1.71 mM CaCl2, 1.19 mM KH2PO4, 1.50 mM glucose, 1.00 mM L-glutamine, 0.49 mM MgCl2, 0.33 mM sodium pyruvate, 2.0X NEAA, 0.5X EAA, 50 μg/ml gentamicin, 5 μg/ml phenol red and 8 mg/ml BSA; pH 7.2 to 7.4) medium at 38.5°C in a CO_2_ incubator (5% CO_2_ in a humidified environment) until 120 h.

### Detection of Intracellular ROS With NBT Staining

The NBT (0.2%) staining solution was prepared in PBS. Nitroblue tetrazolium (20 mg) powder was added to PBS (10 ml), incubated at 37°C for 30 min and vortexed briefly to dissolve. The cumulus expanded matured COC were collected from the control and treatment groups following the 24 h of IVM for the NBT staining. Similarly, for the NBT staining, 8-cell embryos, morula and degenerating embryos with fragmented cell mass were collected at the 72, 120, and 120 h post-culture, respectively. The collected samples were washed sequentially in 3 ml of 4% BSA supplemented PBS (two washes) and 3 ml of NBT staining solution (one wash) in 35 mm culture dishes. The samples were then added to 200 μl drops of NBT staining solution in 35 mm culture dishes (one drop in each dish and 5 COC/embryos in each drop) and the dishes were closed with lids. Subsequently, the culture dishes were kept in an airtight plastic container and tissue papers soaked with sterile water were kept inside the container to maintain humidified environment. The container was then incubated in a CO_2_ incubator at 38.5°C in a humidified atmosphere for 45 min. Following the incubation, the samples were washed in 3 ml of 4% BSA supplemented PBS in 35 mm culture dishes (three washes), placed onto clean glass slides and extra PBS was pipetted off carefully without allowing the samples to dry. A drop of Fluoromount aqueous mounting medium was placed on the samples, and a clean cover slip was placed onto the drop of mounting medium and allowed to be air-dried overnight. Additionally, to prevent zona lysis following mounting, only the embryo samples were fixed after NBT staining in paraformaldehyde (PFA) solution (4% PFA and 4% sucrose in PBS) for 10 min and washed in 3 ml of 4% BSA supplemented PBS in 35 mm culture dishes (three washes) before mounting them on slides. Finally, the stained samples were examined and images (bright-field as well as phase-contrast) were captured using an upright research microscope (Eclipse-80i, Nikon Instruments Corporation, Tokyo, Japan). Similar camera parameters were used for all the experimental groups within an experiment.

### Determining Intracellular ROS Level Following NBT Staining

**FIGURE 1:**
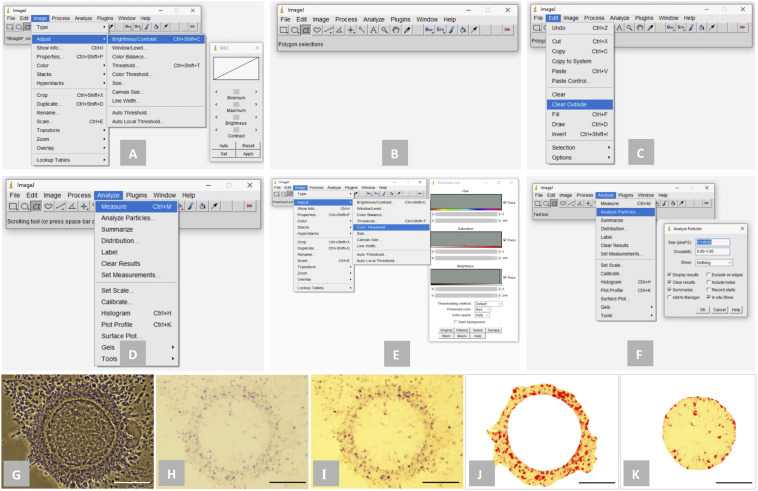
Detection and quantification of the spots of formazan precipitates (dark purple/blue color) using ImageJ 1.52k software. **(A)** “Brightness/Contrast” tool for adjusting brightness and contrast of a bright-field image; **(B)** “Polygon selections” tool for selecting a specific zone; **(C)**: “Clear Outside” tool for clearing outside area beyond the selected zone; **(D)** “Measure” tool for determining the total area of a selected zone; **(E)** “Color Threshold” tool for highlighting the spots of formazan precipitates; **(F)** “Analyze Particles” tool for determining the total area of the highlighted spots of formazan precipitates; **(G)** phase-contrast image of a representative NBT-stained cumulus-oocyte complex (COC); **(H)** bright-field image of the COC; **(I)** bright-field image of the COC following the adjustment of brightness and contrast; **(J)** the zone of compact cumulus cell mass of the COC was selected and the spots of formazan precipitates were highlighted; **(K)** the zone of oocyte of the COC was selected and the spots of formazan precipitates were highlighted. Scale bar = 100 μm.

Following the NBT staining, the intracellular formazan precipitates (dark purple/blue spots) were detected and quantified in the stained oocytes, cumulus cells and embryos using the ImageJ 1.52k software^1^ (NIH, United States; Figure 1). Briefly, brightness and contrast of the bright-field images were adjusted appropriately using the “Brightness/Contrast” tool under the “Image” menu (Image > Adjust > Brightness/Contrast; “Minimum” was adjusted between 10 to 40 and “Maximum” was adjusted between 200 to 230; Figures 1A,I) to visualize the formazan precipitates as clearly as possible. Then, using the “Polygon selections” tool from the toolbar, the zone of oocyte, compact cumulus cell mass or embryo was selected precisely (Figures 1B,J,K). To create the selection, mouse was clicked repeatedly to create line segments and when finished, the small box at the starting point was clicked. The outside area beyond the selected zone was cleared using the “Clear Outside” tool under the “Edit” menu (Edit > Clear Outside; Figures 1C,J,K). Total area of the selected zone (Area-A) was analyzed using the “Measure” tool under the “Analyze” menu (Analyze > Measure; Figure 1D). Thereafter, using the “Color Threshold” tool under the “Image” menu (Image > Adjust > Color Threshold; Figures 1E,J,K), the spots of formazan precipitates were highlighted. Finally, total area of the highlighted spots of formazan precipitates (Area-B) was analyzed using the “Analyze Particles” tool under the “Analyze” menu (Analyze > Analyze Particles; Figure 1F). The level of intracellular ROS was expressed as the proportion (%) of the NBT stained area of oocyte and compact cumulus cell mass (determined from the five individual COC in each experimental group) or embryo (determined from the five individual embryos in each experimental group), which was determined using the equation given below.

Intracellular ROS level (% NBT stained area) = (Area-B/Area-A) × 100.

### Detection of Intracellular ROS With DCFH-DA Staining

Cumulus-oocyte complexes were stained with DCFH-DA to assess the intracellular ROS level according to the method described previously (Javvaji et al., 2019). Briefly, cumulus expanded COC were collected from the control and treatment groups following the 24 h of IVM, washed in PBS and incubated in 10 μM of DCFH-DA in PBS for 30 min in dark. Following the incubation, the COC were washed thoroughly in PBS, placed into the well of a hanging drop slide in 20 μl of PBS and observed under a fluorescent microscope (Eclipse-80i Nikon Instruments Corporation, Tokyo, Japan) using FITC filter (excitation: 485 nm; emission: 535 nm). Fluorescence as well as phase-contrast images of the stained COC (five COC in each experimental group) were captured using the similar camera parameters for all the experimental groups.

### Statistical Analysis

Statistical analyses were performed using the PASW 18.0.0 software package (SPSS/IBM, IL, United States). The values expressed in percentage for the intracellular ROS level were subjected to arcsine transformation to maintain homogeneity of variances and were subjected to Student’s *t*-test to analyze the variations among the experimental groups. All data were presented as mean ± SE and differences were considered significant if the *P*-value was less than 0.05.

## Results

### NBT Staining for Assessing Intracellular ROS in Oocytes and Cumulus Cells

**FIGURE 2:**
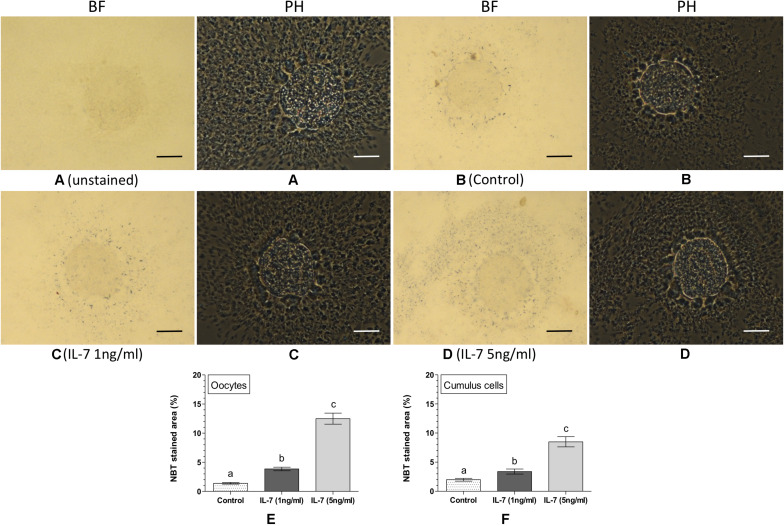
Nitroblue tetrazolium (NBT) staining for assessing intracellular reactive oxygen species (ROS) in oocytes and cumulus cells. Ovine cumulus-oocyte complexes (COC) were matured *in vitro* without (control) or with IL-7 (1 or 5 ng/ml) supplementation and stained with NBT. The dark purple/blue spots of formazan precipitates represent ROS. The level of intracellular ROS (% NBT stained area, mean ± SE) was determined in oocytes and cumulus cells from five individual COC in each experimental group. **(A)** unstained COC, control group; **(B)** NBT-stained COC, control group; **(C,D)** NBT-stained COC, IL-7 supplemented groups; **(E,F)** level of intracellular ROS in oocytes and cumulus cells in different experimental groups. Values without a common superscript (a,b,c) above error bar differ significantly (*P* < 0.05). BF, bright-field image; PH, phase-contrast image. Scale bar = 50 μm.

Intracellular ROS was detected and quantified in the matured oocytes and cumulus cells based on the NBT staining (Figure 2). No formazan precipitate was detected in the unstained COC (Figure 2A). In contrast, NBT staining revealed purple/blue formazan precipitates (representing ROS) in COC, in all the experimental groups (Figures 2B–D). The IL-7 treatments (Figures 2C,D) resulted greater quantity of formazan precipitates in oocytes and cumulus cells as compared to the control (Figure 2B). In oocytes, the level of intracellular ROS (% NBT stained area) was found significantly (*P* < 0.05) lesser in the control (1.39 ± 0.04%) as compared to the 1 ng/ml IL-7 (3.85 ± 0.30%) or 5 ng/ml IL-7 (12.49 ± 0.95%) treatments (Figure 2E). Similarly, the level of intracellular ROS in cumulus cells was found significantly (*P* < 0.05) lesser in the control (1.98 ± 0.24%) than the 1 ng/ml IL-7 (3.39 ± 0.43%) and 5 ng/ml IL-7 (8.49 ± 0.87%) treated groups (Figure 2F). Further, it was evident that the 5 ng/ml IL-7 treatment significantly (*P* < 0.05) stimulated the production of intracellular ROS in both oocytes and cumulus cells as compared to the 1 ng/ml IL-7 treatment (Figures 2E,F).

### NBT Staining for Assessing the Effect of Tempol on Intracellular ROS in Oocytes and Cumulus Cells

**FIGURE 3:**
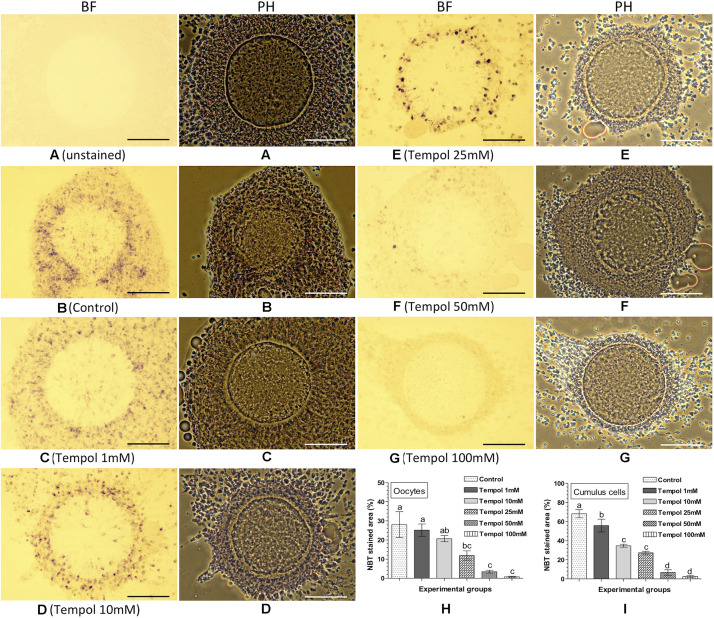
Nitroblue tetrazolium (NBT) staining for assessing the effect of superoxide ion scavenger (Tempol) on intracellular ROS in oocytes and cumulus cells. Ovine cumulus-oocyte complexes (COC) were matured *in vitro* without (control) or with Tempol (1, 10, 25, 50, or 100 mM) supplementation and stained with NBT. The dark purple/blue spots of formazan precipitates represent ROS. The level of intracellular ROS (% NBT stained area, mean ± SE) was determined in oocytes and cumulus cells from five individual COC in each experimental group. **(A)** Unstained COC, control group; **(B)** NBT-stained COC, control group; **(C–G)** NBT-stained COC, Tempol supplemented groups; **(H,I)** level of intracellular ROS in oocytes and cumulus cells in different experimental groups. Values without a common superscript (a,b,c,d) above error bar differ significantly (*P* < 0.05). BF, bright-field image; PH, phase-contrast image. Scale bar = 100 μm.

The effect of the supplementation of Tempol (superoxide ion scavenger) on intracellular ROS in the matured oocytes and cumulus cells was assessed based on the NBT staining (Figure 3). No formazan precipitate was detected in the unstained COC (Figure 3A). The NBT staining revealed that as compared to the control (Figure 3B), the supplementation of Tempol reduced the quantity of formazan precipitates (representing ROS) in oocytes and cumulus cells in a dose dependent manner (Figures 3C–G). Evidently the 100 mM Tempol treatment resulted the lowest level (*P* < 0.05) of intracellular ROS (% NBT stained area) in oocytes (0.74 ± 0.28%) and cumulus cells (2.50 ± 1.06%) as compared to the control (28.1 ± 6.70% in oocytes and 68.1 ± 4.24% in cumulus cells) group (Figures 3H,I).

### NBT Staining for Assessing the Combined Effect of IL-7 and Tempol on Intracellular ROS in Oocytes and Cumulus Cells

**FIGURE 4:**
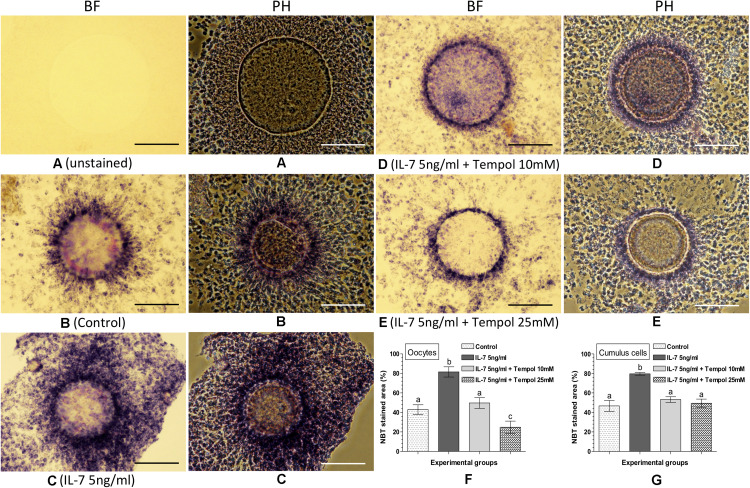
Nitroblue tetrazolium (NBT) staining for assessing the combined effect of Interleukin-7 (IL-7) and superoxide ion scavenger (Tempol) on intracellular ROS in oocytes and cumulus cells. Ovine cumulus-oocyte complexes (COC) were matured *in vitro* without (control) or with the supplementation of IL-7 (5 ng/ml) or combination of IL-7 (5 ng/ml) and Tempol (10 or 25 mM) and stained with NBT. The dark purple/blue spots of formazan precipitates represent ROS. The level of intracellular ROS (% NBT stained area, mean ± SE) was determined in oocytes and cumulus cells from five individual COC in each experimental group. **(A)** Unstained COC, IL-7 supplemented group; **(B)** NBT-stained COC, control group; **(C)** NBT-stained COC, IL-7 supplemented group; **(D,E)** NBT-stained COC, combined supplementation (IL-7 + Tempol) groups; **(F,G)** level of intracellular ROS in oocytes and cumulus cells in different experimental groups. Values without a common superscript (a,b,c) above error bar differ significantly (*P* < 0.05). BF, bright-field image; PH, phase-contrast image. Scale bar = 100 μm.

The effect of the combined supplementation of IL-7 and Tempol on intracellular ROS in the matured oocytes and cumulus cells was assessed based on the NBT staining (Figure 4). No formazan precipitate was detected in the unstained COC (Figure 4A). The NBT staining revealed that the combined supplementation of 5 ng/ml IL-7 along with 10 or 25 mM Tempol resulted lesser quantity of formazan precipitates (representing ROS) in oocytes as well as cumulus cells (Figures 4D,E) as compared to the supplementation of 5 ng/ml IL-7 alone (Figure 4C). The level of intracellular ROS (% NBT stained area) was found significantly (*P* < 0.05) greater in oocytes (81.5 ± 5.27%) and cumulus cells (79.6 ± 1.35%) in the 5 ng/ml IL-7 treated group as compared to the 5 ng/ml IL-7 and 10 mM Tempol treated (49.7 ± 5.48 and 53.3 ± 2.86%, respectively), 5 ng/ml IL-7 and 25 mM Tempol treated (24.8 ± 6.30 and 49.5 ± 4.21%, respectively) and control (42.9 ± 4.96 and 46.7 ± 5.58%, respectively) groups (Figures 4F,G).

### DCFH-DA Staining for Assessing Intracellular ROS in Cumulus-Oocyte Complexes

**FIGURE 5:**
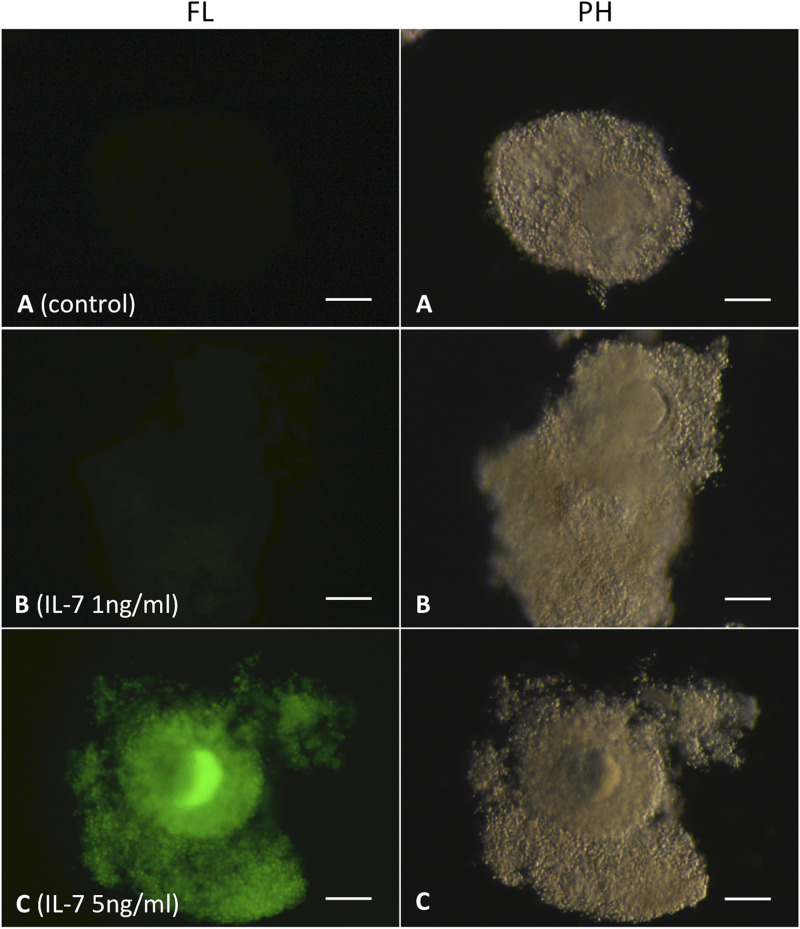
Assessment of intracellular ROS in cumulus-oocyte complexes (COC) based on 2′, 7′-dichlorodihydrofluorescein diacetate (DCFH-DA) staining. Ovine COC were matured *in vitro* without (control) or with IL-7 (1 or 5 ng/ml) supplementation and stained with DCFH-DA. The green color DCF fluorescence represents ROS. **(A)** COC, control group; **(B,C)** COC, IL-7 supplemented groups. Five COC were assessed in each experimental group. FL, fluorescence image; PH, phase-contrast image. Scale bar = 100 μm.

The level of intracellular ROS in the matured COC was also confirmed based on the DCFH-DA staining (Figure 5). It was evident that the IL-7 treatments (1 and 5 ng/ml) increased the production of intracellular ROS in the matured COC. The intensity of DCF fluorescence (representing intracellular ROS) in the COC of control group (Figure 5A) was found negligible as compared to that of the IL-7 treated groups (Figures 5B,C). Further, the most intense DCF fluorescence in the 5 ng/ml IL-7 treated group indicated that the level of intracellular ROS was highest in this experimental group (Figure 5C).

### NBT Staining for Assessing Intracellular ROS in Embryos

**FIGURE 6:**
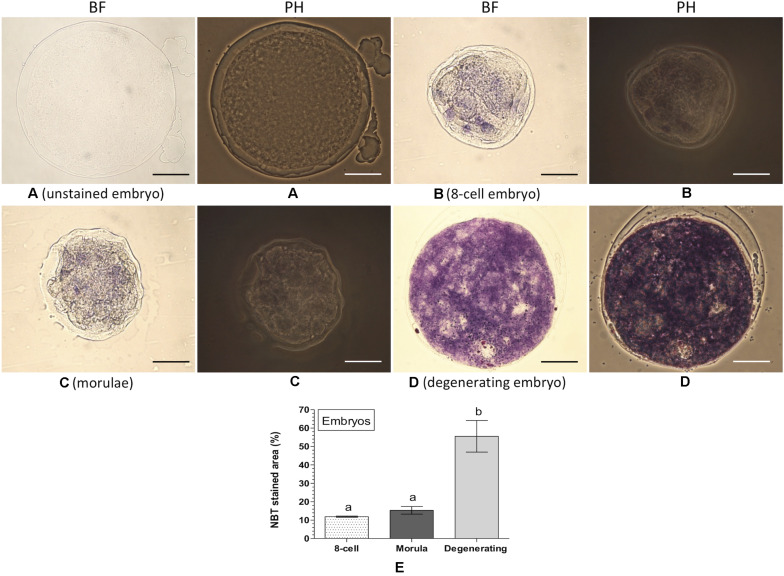
Nitroblue tetrazolium (NBT) staining for assessing intracellular ROS in embryos. Ovine embryos (8-cell, morula and degenerating) were produced *in vitro* and stained with NBT. The dark purple/blue spots of formazan precipitates represent ROS. The level of intracellular ROS (% NBT stained area, mean ± SE) was determined in five individual embryos at each developmental stage. **(A)** Unstained degenerating embryo; **(B)** NBT-stained 8-cell embryo; **(C)** NBT-stained morulae; **(D)** NBT-stained degenerating embryo; **(E)** level of intracellular ROS in embryos at different developmental stages. Values without a common superscript (a,b) above error bar differ significantly (*P* < 0.05). BF, bright-field image; PH, phase-contrast image. Scale bar = 50 μm.

Intracellular ROS was also detected and quantified in embryos at the different developmental stages based on the NBT staining (Figure 6). No formazan precipitate was detected in the unstained degenerating embryos (Figure 6A). In contrast, NBT staining revealed purple/blue formazan precipitates (representing ROS) in the 8-cell embryos (Figure 6B), morula (Figure 6C), and degenerating embryos (Figure 6D). The level of intracellular ROS (% NBT stained area) did not differ significantly between the 8-cell embryos (11.9 ± 0.35%) and morula (15.3 ± 2.07%). In contrast, significantly (*P* < 0.05) greater level of intracellular ROS (55.5 ± 8.56%) was noticed in the degenerating embryos as compared to that of the 8-cell embryos or morula (Figure 6E).

## Discussion

Reactive oxygen species contribute to the regulation of ovulation, oocyte maturation fertilization and embryo development. Therefore, assessing the level of intracellular ROS is important for evaluating the developmental capability and cellular functions of oocytes, cumulus cells and embryos. In this study, we report an efficient method for detecting and quantifying intracellular ROS in oocytes, cumulus cells and embryos, based on the NBT staining and bright-field microscopy.

Although, the fluorescence-based DCFH-DA staining is the widely used method for detecting intracellular ROS in oocytes and embryos (Bae, 2015; Mihalas et al., 2017; Song et al., 2017; Yang et al., 2018), several limitations are associated with the method (Choi et al., 2012; Kalyanaraman et al., 2012; Roesslein et al., 2013). Therefore, in this study, we developed an efficient and easy-to-perform alternative method for detecting and quantifying intracellular ROS in oocytes, cumulus cells and embryos. This method is based on NBT staining and bright-field microscopy. In this method, NBT enters into oocytes, cumulus cells and embryos following their incubation in NBT solution. Subsequently, within the cells, NBT reacts with superoxide ions and forms intracellular purple/blue water insoluble and stable formazan precipitates. These precipitates can be detected and quantified using bright-field microscopy and ImageJ software.

Superoxide anion is a major species of ROS. It is the precursor of most other ROS molecules and a mediator in the oxidative chain reactions (Turrens, 2003; Chen et al., 2009; Gosalvez et al., 2017). Therefore, the assessment of intracellular superoxide level using the developed method can indirectly reflect the overall status of intracellular ROS. It has been demonstrated previously that NBT staining is an effective method for detecting intracellular ROS as the levels of ROS assessed by chemiluminescence assay are strongly correlated with the results of NBT staining (Esfandiari et al., 2003).

The NBT staining has been used previously for detecting ROS in different mammalian cell types such as retina (Zhang et al., 1993), adipocytes and macrophages (Lagathu et al., 2007), leukocytes (Esfandiari et al., 2003) and spermatozoa (Esfandiari et al., 2003; Tunc et al., 2010). Nevertheless, at present, no report indicates the use of NBT staining for detecting or quantifying intracellular ROS in oocytes, cumulus cells or embryos. In this study, we demonstrated that the NBT staining could be used effectively to detect and quantify intracellular ROS in oocytes, cumulus cells and embryos. Ovine COC were treated with IL-7 at two different levels (1 and 5 ng/ml) during IVM to stimulate the generation of intracellular ROS in oocytes and cumulus cells (Javvaji et al., 2019). As expected, significantly greater proportion of formazan precipitates was observed in the matured oocytes and cumulus cells in the 5 ng/ml IL-7 treated group as compared to the control or 1 ng/ml IL-7 treatment. The results indicated a significant increase in the level of intracellular ROS in oocytes and cumulus cells following the treatment with 5 ng/ml of IL-7. The observation was confirmed as well based on the DCFH-DA staining of the ovine matured COC.

Comparison of DCF fluorescence intensity is difficult if there is a wide difference in the level of intracellular ROS among the experimental groups. In general, for the assessment of ROS level, fluorescence signal in the different experimental groups is documented using the similar camera parameters that are adjusted for the samples emitting intense fluorescence. As a result, the samples with low fluorescence intensity appear extremely dark, resulting poor visual representation of ROS within the cells. This phenomenon was also evident in our study. The DCF fluorescence signal was hardly visible in the COC of control group with low level of intracellular ROS. In contrast, in the same experimental group, the NBT staining resulted better visual perception of intracellular ROS with clear visibility of the formazan precipitates within the cells.

It may be noted that cellular oxidoreductases can also reduce NBT and form formazan precipitates using NADH or NADPH as an electron donor (Baehner et al., 1976). Therefore, in this study, we also evaluated the extent of non-specific reduction of NBT in oocytes and cumulus cells by molecules other than superoxide ions. Previous report indicates that the superoxide ion scavenger Tempol effectively neutralizes intracellular superoxides in rat vascular smooth muscle cells (Luo et al., 2009) and human prostate cancer cells (Lejeune et al., 2006). In the current study, ovine COC were treated with different concentrations of Tempol to neutralize intracellular superoxides. It was evident that the quantity of formazan precipitates decreased in the treated oocytes and cumulus cells with an increase in the concentration of Tempol. The least proportion of NBT stained area (representing the level of intracellular ROS) was found in the oocytes and cumulus cells following the treatment with 100 mM of Tempol (less than 5% as compared to the control). If it is assumed that the entire amount of intracellular superoxides was neutralized with the 100 mM Tempol treatment, then the magnitude of non-specific NBT reduction by other molecules was negligible. The results indicate that the developed NBT staining method is precise for detecting and interpreting the level of intracellular ROS.

We further validated the ability of IL-7 to stimulate the generation of intracellular ROS in oocytes and cumulus cells based on the combined treatments of IL-7 and superoxide scavenger (Tempol) and NBT staining. Ovine COC were treated with the combination of IL-7 (5 ng/ml) and Tempol (10 or 25 mM) during IVM and the matured COC were stained with NBT. It was evident that the combined treatments of IL-7 and Tempol resulted significantly lesser proportion of NBT stained area in oocytes and cumulus cells as compared to the treatment of IL-7 alone. Further, the proportions of NBT stained area in oocytes and cumulus cells were found similar or lesser in the combined treatment groups as compared to that of the control. These results confirmed that IL-7 stimulated the production of intracellular ROS, but not in the presence of superoxide scavenger.

We also detected and quantified intracellular ROS in ovine embryos at different developmental stages, based on the NBT staining. The production of intracellular ROS is a distinctive attribute of embryo metabolism (Hajian et al., 2017). The ROS-mediated oxidative stress during IVC results poor embryo quality and viability (Truong et al., 2016), and antioxidant treatment during IVF and IVC significantly improves embryo development (Truong and Gardner, 2017). It is also evident that the fragmented embryos contain greater concentration of intracellular ROS that has been linked to apoptosis (Yang et al., 1998). In the current study, the level of intracellular ROS did not differ significantly between the 8-cell embryos and morula. In contrast, as expected, the intracellular ROS level was found significantly greater in the degenerating embryos as compared to the 8-cell embryos or morula.

## Conclusion

An efficient method has been standardized for detecting and quantifying intracellular ROS in oocytes, cumulus cells and embryos, based on the NBT staining and bright-field microscopy. The developed method is easy-to-perform and allows good visual representation and interpretation of the level of intracellular ROS in individual COC or embryo. However, the developed method allows detection and quantification of intracellular superoxides and provides an indirect measurement of the overall status of intracellular ROS. Further, although negligible, the method can lead to measurement errors due to the non-specific reduction of NBT by molecules other than superoxides. Nevertheless, the current study reveals that the developed NBT staining method is a suitable alternative to the DCFH-DA staining for interpreting the level of intracellular ROS in oocytes, cumulus cells and embryos. This newly developed method would be a useful tool for the developmental biologists for assessing intracellular ROS in individual COC or embryo.

## Data Availability Statement

All datasets generated for this study are included in the article.

## Ethics Statement

The animal study was reviewed and approved, and all experiments including the use of ram for semen collection met the guidelines and regulations of the Institutional Animal Ethics Committee (IAEC) of the ICAR-National Institute of Animal Nutrition and Physiology (ICAR-NIANP), Bengaluru, India.

## Author Contributions

AD, AK, and PJ conceived the study. PJ, AD, and JF conducted the experiments. PJ and AD wrote the manuscript. All authors analyzed the data and revised final version of the manuscript.

## Conflict of Interest

The authors declare that the research was conducted in the absence of any commercial or financial relationships that could be construed as a potential conflict of interest.

## Footnotes

1 https://imagej.nih.gov/ij
